# Stem cell protein Piwil1 endowed endometrial cancer cells with stem-like properties via inducing epithelial-mesenchymal transition

**DOI:** 10.1186/s12885-015-1794-8

**Published:** 2015-10-27

**Authors:** Zheng Chen, Qi Che, Xiaoying He, Fangyuan Wang, Huihui Wang, Minjiao Zhu, Jing Sun, Xiaoping Wan

**Affiliations:** 1 Department of Obstetrics and Gynecology, Shanghai First People’s Hospital Affiliated to Shanghai Jiao Tong University, Shanghai, China; 2 Department of Obstetrics and Gynecology, International Peace Maternity and Child Health Hospital Affiliated to Shanghai Jiao Tong University School of Medicine, Shanghai, China; 3 Department of Obstetrics and Gynecology, Shanghai First Maternity and Infant Hospital, Tong Ji University School of Medicine, No. 536, Changle Road, Shanghai, 200080 China

**Keywords:** Piwil1, Stem-like properties, EMT, Endometrial cancer

## Abstract

**Background:**

Stem cell protein Piwil1 functions as an oncogene in various tumor types. However, the exact function and mechanism of Piwil1 in endometrial cancer remains unclear.

**Methods:**

The expression of Piwil1 and its relationships with clinicopathological factors were investigated using immunohistochemistry. Up- or down-regulation of Piwil1 were achieved by stable or transient transfection with plasmids or short hairpin RNA (shRNA). Effects of Piwil1 on cancer cells viability, invasion and migration were evaluated by MTT, plate colony formation, transwell assay and nude mouse tumor xenograft assay. The stem-like properties of endometrial cancer cells was detected by spheroid formation assay. Effects of Piwil1 on expression levels of target genes were detected by qRT-PCR, western blotting and Immunofluorescence.

**Results:**

Compared with atypical hyperplasia and normal tissues, Piwil1 was much higher in endometrial carcinoma tissues. We found that Piwil1 expression was significantly correlated with FIGO stage, lymphovascular space involvement, lymph node metastasis and level of myometrial invasion. Overexpression of Piwil1 functioned to maintain stem-like characteristics, including enhancing tumor cell viability, migration, invasion and sphere-forming activity. Conversely, Piwil1 knockdown inhibited cell viability, migration, invasion, sphere-forming activity in vitro and tumor formation in xenograft model in vivo. Furthermore, study of the expression of epithelial and mesenchymal markers showed that Piwil1 was responsible for an EMT-like phenotype associated with an increase in mesenchymal markers and suppression of E-cadherin. Moreover, Piwil1 augmented expression levels of CD44 and ALDH1 expression, two known endometrial CSC markers, as well as other stemness-associated genes.

**Conclusions:**

Our results suggested that stem cell protein Piwil1 play important roles in regulating EMT and the acquisition of stem-like properties of endometrial cancer cells. Therefore, it indicated that Piwil1 may represent a promising target for developing a novel treatment strategy for endometrial cancer.

**Electronic supplementary material:**

The online version of this article (doi:10.1186/s12885-015-1794-8) contains supplementary material, which is available to authorized users.

## Background

Cancer of the corpus uteri (commonly called endometrial cancer) is one of the most common malignancies of the reproductive system. In the United States, approximately 52,630 new cases will be diagnosed in 2014 and 8590 deaths are expected [1]. Therefore, it is critical to better understand the molecular mechanisms of endometrial cancer.

Cancer stem cells (CSCs) are defined by their ability to seed new tumors and are proposed to be the cancer-initiating cells responsible for carcinogenesis [2, 3]. To date, CSCs have been isolated from various human cancer tissues [4, 5]. Emerging evidence indicates that a population of CSCs may be involved in endometrial carcinoma carcinogenesis [6, 7]. The epithelial-mesenchymal transition (EMT) is a well-described process whereby epithelial cells lose their polarity and cell-cell contacts, undergoing a dramatic remodeling of the cytoskeleton and acquiring a migratory phenotype. Recent studies have highlighted a link between EMT and the induction of the stem cell-like properties of cancer cells in solid tumors [8–10].

The Piwil1 gene is a member of the Piwi gene family, which represent the first class of evolutionarily conserved genes known to be required for stem cell self-renewal and division [11, 12]. Piwil1 has been found to be frequently overexpressed in various tumor types, including gastric cancer, breast cancer and endometrial cancer [13, 14]. Previous studies have demonstrated that Piwil1 played a key role in enhancing tumor malignant behavior including proliferation [13, 15] and invasion [16], as well as the potential importance of Piwil1 expression as a marker of poor prognosis in soft-tissue sarcoma and ductal adenocarcinoma of the pancreas [17, 18].

On the basis of these findings, we believe that both or partly of Piwil1 and EMT are involved in the acquisition of stem-like properties, with the hope that such associations might provide insights into the causal function of Piwil1 in endometrial carcinogenesis.

## Methods

### Ethics statement

The study was approved by the Human Investigation Ethics Committee of Shanghai First People’s Hospital Affiliated to Shanghai Jiao Tong University. The samples of endometrial carcinoma and normal endometrial tissues were collected after written informed consent from the patients. Animal research was carried out in strict accordance with the Guide for the Care and Use of Laboratory Animals. The procedures were approved by the Department of Laboratory Animal Science at Shanghai Jiao Tong University School of Medicine. All efforts were made to minimize suffering.

### Tissue specimens

Tissue samples for immunohistochemistry and real-time quantity PCR (RT-qPCR) were obtained at Shanghai First People’s Hospital Affiliated to Shanghai Jiao Tong University from 2011 to 2013. The stages and histological grades of these tumors were established according to the criteria of the Federation International of Gynecology and Obstetrics (FIGO) surgical staging system (2009) [19]. None of the patients underwent hormone therapy, radiotherapy, or chemotherapy prior to surgery.

### Cell culture

Human endometrial cancer cell lines including Ishikawa and HEC-1B were obtained from the Chinese Academy of Sciences Committee Type Culture Collection (Shanghai, China, Additional file 3). According to the provider’s instructions, cells were cultured at 37 °C in a humidified atmosphere containing 5 % CO_2_ in Dulbecco’s modified Eagle’s medium (DMEM)/F12 (Gibco, Life Technologies, Auckland, New Zealand) supplemented with 10 % fetal bovine serum (Gibco, Carlsbad, CA, USA).

Ishikawa cell line is a human endometrial adenocarcinoma cell line which contains estrogen and progesterone receptors [20]. HEC-1B cell line is a human endometrial adenocarcinoma cell line which has a low baseline level of estrogen and progesterone receptors [21].

### Immunohistochemistry

Tissue immunohistochemistry was performed by the 3,3'-diaminobenzidine (DAB) method with a heat-induced antigen retrieval step. Briefly, slides were incubated with rabbit polyclonal anti-Piwil1 (1:100, ab105393, Abcam), rabbit monoclonal anti-E-cadherin (1:400, #3195, CST), rabbit monoclonal anti- Vimentin (1:100, #5741, CST), rabbit monoclonal anti-CD44 (1:100, ab51037, Abcam), rabbit monoclonal anti-ALDH1 (1:100, ab52492, Abcam), rabbit monoclonal anti-ki67 (1:100, ab16667, Abcam) and rabbit monoclonal anti-PCNA (1:100, ab92552, Abcam) overnight at 4 °C and then incubated with horseradish peroxidase (HRP)-linked anti-rabbit or anti-mouse secondary antibody (Boster) at room temperature for 30 min followed by chromagen detection with DAB (Boster) and hematoxylin (Boster) counterstaining. Isotype control antibodies was used as negative control.

Two independent pathologists, who were blinded to the clinical and pathological data, evaluated the specimens. Sections were evaluated according to semi quantitative immunoreactivity scores. We separately scored for the percentage of positive staining (0 = negative, 1 = 25 %, 2 = 25–50 %, 3 = 50–75 % and 4 = 75 %) and the staining intensity (0 = none, 1 = weak, 2 = moderate, and 3 = strong). For each specimen, the summation of the two above gave the final score.

### Total RNA extraction and real-time RT-PCR

Total RNA was extracted from tissues and cell lines using Trizol (Invitrogen) and cDNA was prepared using the reverse transcriptase kit (TaKaRa) according to the manufacturer’s instructions. The cDNA was analyzed by real-time PCR using SYBR Premix Ex Taq (TaKaRa) in an Eppendorf Mastercycler realplex. A housekeeping gene, GAPDH, was used as an internal control. Data was calculated using the 2^-△△Ct^ formula. Primers sequences are shown in Additional file 1: Table S1.

### Western blotting

Cells were lysed in lysis buffer (Beyotime) for 30 min at 4 °C. Total proteins were fractionated by SDS–PAGE and transferred onto PVDF membranes (Millipore). The membranes were then incubated with primary antibodies against Piwil1 (1:1000, ab105393, Abcam), E-cadherin (1:1000, #3195, CST), N-cadherin (1:1000, #13116, CST), Vimentin (1:1000, #5741, CST), CD44 (1:5000, ab51037, Abcam), CD133 (1:1000, 18470-1-AP, ProteinTech) and ALDH1 (1:1000, ab52492, Abcam) at 4 °C overnight, followed by incubation with peroxidase-linked secondary antibody (1:10000, 112-005-003, Jackson ImmunoResearch). The probed proteins were detected by enhanced chemiluminescent reagents (Thermo). GAPDH (1:2000, #5174, CST) was used as an internal control.

### Immunofluorescence

Cells were cultured on glass coverslips for 24 h and then fixed in 4 % paraformaldehyde. They were permeabilized with 0.1 % Triton X-100. After blocking in 5 % bovine serum albumin for 1 h at room temperature, cells were incubated with primary antibodies as follows: Piwil1 (1:50, ab105393, Abcam), E-cadherin (1:200, #3195, CST), Vimentin (1:100, #5741, CST), CD44 (1:100, ab51037, Abcam), CD133 (1:100, 18470-1-AP, ProteinTech) and ALDH1 (1:1000, ab52492, Abcam) overnight at 4 °C. Next, cells were incubated with Alexa Fluor 647 or rhodamine (TRITC)-conjugated secondary antibodies (1:200, Jackson ImmunoResearch) for 1 h. Nuclei were visualized by counterstaining with 496-diamidino-2-phenylindole (DAPI). Samples were analyzed using a Leica TCS SP8 confocal microscope (Leica Microsystems). Isotype control antibodies was used as negative control.

### Stable transfection

HEC-1B cells were transfected with Piwil1 expression plasmids (exPiwil1, Genepharma, Shanghai, China) or control plasmids (pEGFP-N1, empty vector, EV, Genepharma) by Lipofectamine^TM^ 2000 (Invitrogen) according to the manufacturer’s protocol. Stable overexpression clones (HEC-1B^exPiwil1^ and HEC-1B^EV^ cells) were selected in the presence of 1 mg/ml G418 (Gibco) and then propagated in the presence of 0.5 mg/ml G418.

Ishikawa cells were transfected with shRNA against Piwil1 (shPiwil1, Genepharma) (sense: 5’-AGTCAGCAACCTGGTTATA-3’; antisense: 5’- TATAACCAGGTTGCTGACTGG -3’) or shRNA against nontarget (NT, Genepharma) by Lipofectamine™ 2000. Stable knockdown clones (Ishikawa^EV^ and Ishikawa^shPiwil1^ cells) were selected in the presence of 0.5 μg/ml puromycin (Sigma; St. Louis, MO, USA) and maintained with 0.3 μg/ml. Transfection efficiency was confirmed by RT-qPCR and western blot.

### MTT and colony-formation assays

Cells and transfected cells (3 × 10^3^ cells/well) were plated in 96-well plates. Then, 20 μl of 3-(4,5-dimethylthiazol-2-yl)-2,5-diphenyl-tetrazolium bromide (MTT, 5 mg/ml; Sigma) was added to each well and then incubated at 37 °C for 4 h. Absorbance values were then measured at 490 nm using a microplate reader (Bio-Red). For colony formation assay, 200 cells/well were seeded into 6-well plates. When clearly identifiable cell clones had formed, the colonies were fixed with methanol and stained with 0.5 % crystal violet. All experiments were repeated at least three times.

### Cell migration and invasion assays

Cell lines were suspended in serum-free medium and plated at a density of 1 × 10^5^ cells/well (for the migration assay) or 2 × 10^5^ cells/well (for the invasion assay) in 6.5 mm transwell chambers equipped with 8.0 μm pore-size polycarbonate membranes without or with matrigel coating (BD Biosciences). Complete medium (600 μl) was added to the lower chamber. After incubation for 24 h (migration assay) or 48 h (invasion assay), cells were fixed in 4 % paraformaldehyde and stained with crystal violet. Then cells that migrated to the basal side of the membrane were counted at 200× magnification. The migration and invasion assays were repeated at least three times.

### Spheroid formation

Cells (5 × 10^3^) were plated on non-adherent 6-well culture plates (coated with a 10 % polyHEMA (Sigma) for 4 h and dried for 3 days at 37 °C). After plating, cells were incubated in a serum-free medium consisting of DMEM/F12 supplemented with 20 ng/ml of EGF, 10 ng/ml of bFGF, and 2 % B27 (all from Sigma and Gibco). The number of spheroids per well was counted after 5 days under light microscopy at a 200-fold magnification. The experiments were repeated at least three times.

### Nude mouse tumor xenograft assay

Athymic female nude mice (BALB/c, 4 to 6 week old, *n* = 5 per group) were obtained from Shanghai Life Science Institute (Slac Laboratory Animal Co., Ltd, Shanghai, China). Ishikawa^shPiwil1^ or Ishikawa^NT^ cells were injected subcutaneously into the flank of each mouse at a density of 1 × 10^7^ cells to establish a mouse model bearing endometrial cancer. The growth of tumors was monitored throughout the experiment and tumor size was measured with calipers every 4 days and the tumor volume was calculated as (Rmax) × (R^2^ min)/2. Four weeks after injection, mice were euthanized, tumors were removed carefully, and the weight and volume of tumors were measured.

### Statistical analysis

All data analyses were performed using the software package SPSS v. 18 (SPSS Inc., Chicago, IL, USA). Values were expressed as mean ± the standard deviation and analyzed with the Student’s *t*-test or Mann–Whitney *U* test. Significant differences were indicated for *P* values < 0.05.

## Results

### Piwil1 was overexpressed in endometrial cancer tissues

**Fig. 1:**
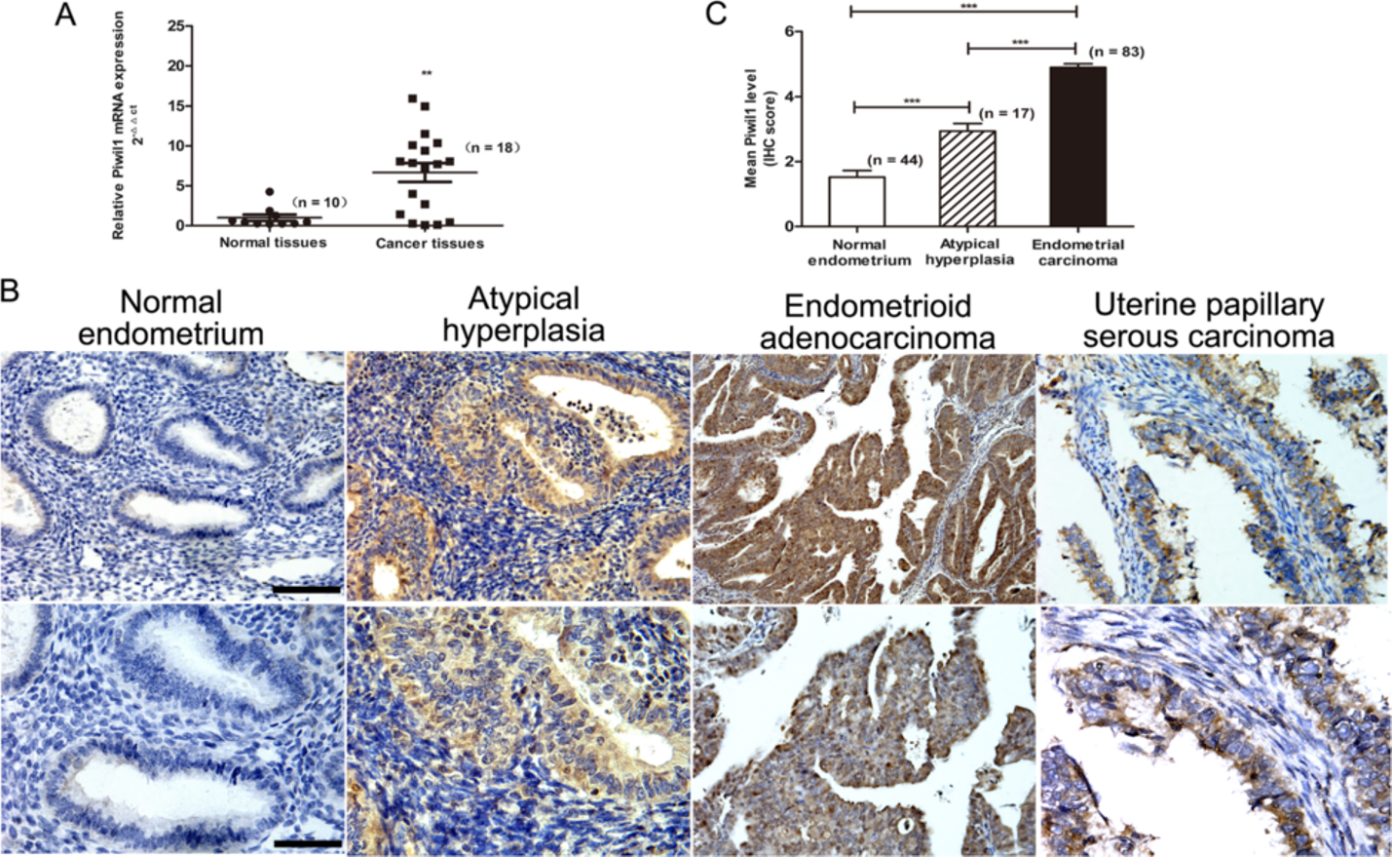
Expression of Piwil1 in human endometrial carcinoma tissue. **a** RT-qPCR analysis for Piwil1 mRNA in normal human endometrial tissues (*n* = 10) and endometrial cancer tissues (*n* = 18) (**P < 0.01). **b** Representative Piwil1 immunohistochemical staining of normal endometrial tissues, endometrial atypical hyperplasia and two endometrial cancer types. Original magnification 200×, scale bar, 100 μm (upper); 400×, scale bar, 50 μm (lower). **c** Immunohistochemistry scores (IS) of Piwil1 in normal endometrium (*n* = 44), atypical hyperplasia (*n* = 17), and endometrial carcinoma tissues (*n* = 83) (***P < 0.001)

We examined 18 endometrial cancer tissues (15 endometrioid and 3 serous) and 10 normal endometrial tissues (6 proliferative and 4 secretory) through RT–qPCR. We observed a striking pattern of Piwil1 overexpression in endometrial cancer tissues compared to normal endometrial tissues (**P < 0.01, Fig. 1a).

To confirm this result at the protein level, we examined the protein expression of Piwil1 in clinical samples using immunohistochemistry. Piwil1 immunostaining was absent or weak in normal endometrial tissues but moderate-to-strong in endometrial atypical hyperplasia and endometrial cancer tissues (Fig. 1b). Consistent with our RT-qPCR analysis, the protein expression of Piwil1 was also significantly higher in endometrial atypical hyperplasia (IS = 2.9, ***P < 0.001) and endometrial cancer tissues (IS = 5.0, ***P < 0.001) compared with normal endometrial tissues (IS = 1.5, Fig. 1c).

We further investigated the association between Piwil1 expression and clinicopathological features in endometrial cancer patients. These analyses showed that increased Piwil1 expression was significantly associated with FIGO stage (*P* = 0.042), lymphovascular space involvement (*P* = 0.000), lymph node metastasis (*P* = 0.026), and level of myometrial invasion (*P* = 0.048), but not with patient age or histological grade (Table 1).

**Table 1:** The correlation of Piwil1 expression with clinicopathological features in endometrial cancer

	Cases (n)	Piwil1 IS	
		Mean ± SD	*P* value
Age			
≤55	34	4.9 ± 1.1	
>55	49	4.9 ± 1.0	0.9
FIGO stage			
I – II	78	4.9 ± 1.0	
III - IV	5	6.4 ± 0.9	0.005
Histological grade			
G1 - G2	73	4.9 ± 1.1	
G3	10	5.5 ± 0.8	0.11
Lymphovascular space involvement			
No	68	4.8 ± 1.0	
Yes	15	5.9 ± 0.8	0.00
Lymph node metastasis			
No	79	4.9 ± 1.1	
Yes	4	6.3 ± 1.0	0.03
Myometrial invasion			
≤1/2	70	4.9 ± 1.0	
>1/2	13	5.7 ± 1.2	0.02

Significance of difference (*P* value) between categories was analyzed by Mann–Whitney *U* test. *P* < 0.05 for the significance of difference

### Overexpression or knockdown of Piwil1 in human endometrial cancer cell lines

RT-qPCR, western blot and immunofluorescence were performed to assess the expression of Piwil1 in endometrial cancer cell lines (Additional file 2: Figure S1). Variable levels of Piwil1 were detected across the endometrial cancer cell lines. Ishikawa (high expression) and HEC-1B (low expression) cell lines were chosen for further experimentation based on their differential expression of Piwil1.

**Fig. 2:**
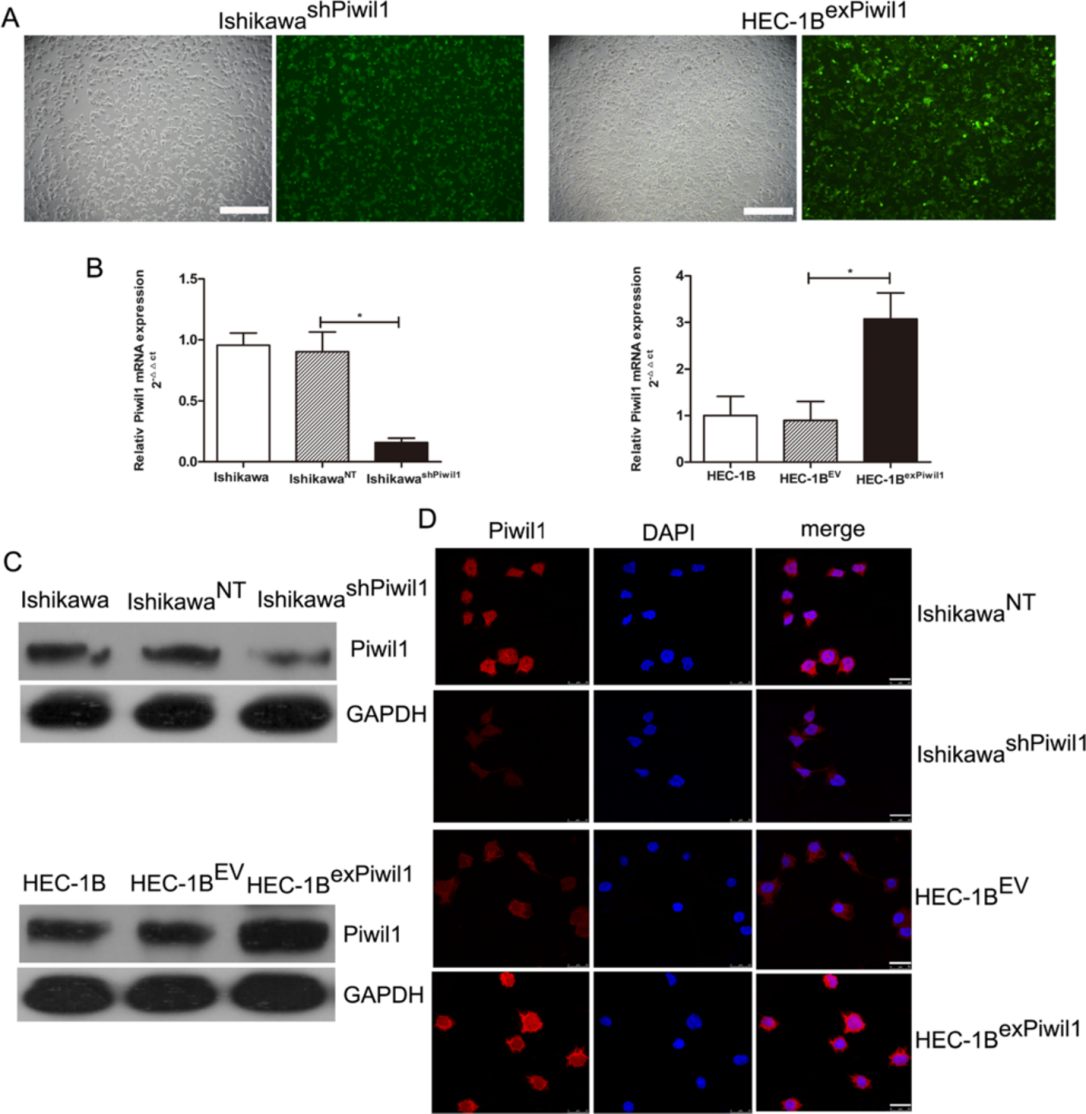
Overexpression or knockdown of Piwil1 in human endometrial cancer cell lines. **a** Stable transfection of Ishikawa cells with shRNA against Piwil1 and HEC-1B cells with Piwil1 expression plasmids. The percentage of transfected cells with fluorescence was > 95 %. (**b** and **c**) RT-qPCR and western blot demonstrated expression level of Piwil1 in Ishikawa, Ishikawa^NT^ and Ishikawa^shPiwil1^ cells or HEC-1B, HEC-1B^EV^ and HEC-1B^exPiwil1^ cells (*P < 0.05). **d** Representative immunofluorescence images showing Piwil1 expression in Ishikawa^NT^ and Ishikawa^shPiwil1^ cells or in HEC-1B^EV^ and HEC-1B^exPiwil1^ cells. Nuclei were stained with DAPI. Scale bars, 25 μm

To determine whether Piwil1 could enhance the stemness of endometrial cancer cells by inducing EMT, we transfected Piwil1 expression plasmids into HEC-1B cell lines to originate Piwil1 overexpression cells (HEC-1B^exPiwil1^ cells) and transfected shRNA against Piwil1 to Ishikawa cell lines to originate Piwil1 knock-down cells (Ishikawa^shPiwil1^ cells). The transfection efficiency was up to 95 % (Fig. 2a). As shown in Fig. 2, HEC-1B^exPiwil1^ cells transfected with Piwil1 expression plasmids significantly increased Piwil1 expression in both mRNA and protein levels compared with HEC-1B^EV^ cell lines (*P < 0.05) and Ishikawa^shPiwil1^ cells transfected with shRNA against Piwil1 significantly decreased Piwil1 expression in both mRNA and protein levels compared with the control cell lines (*P < 0.05).

### Piwil1 led to increased acquisition of endometrial cancer stem cell markers

**Fig. 3:**
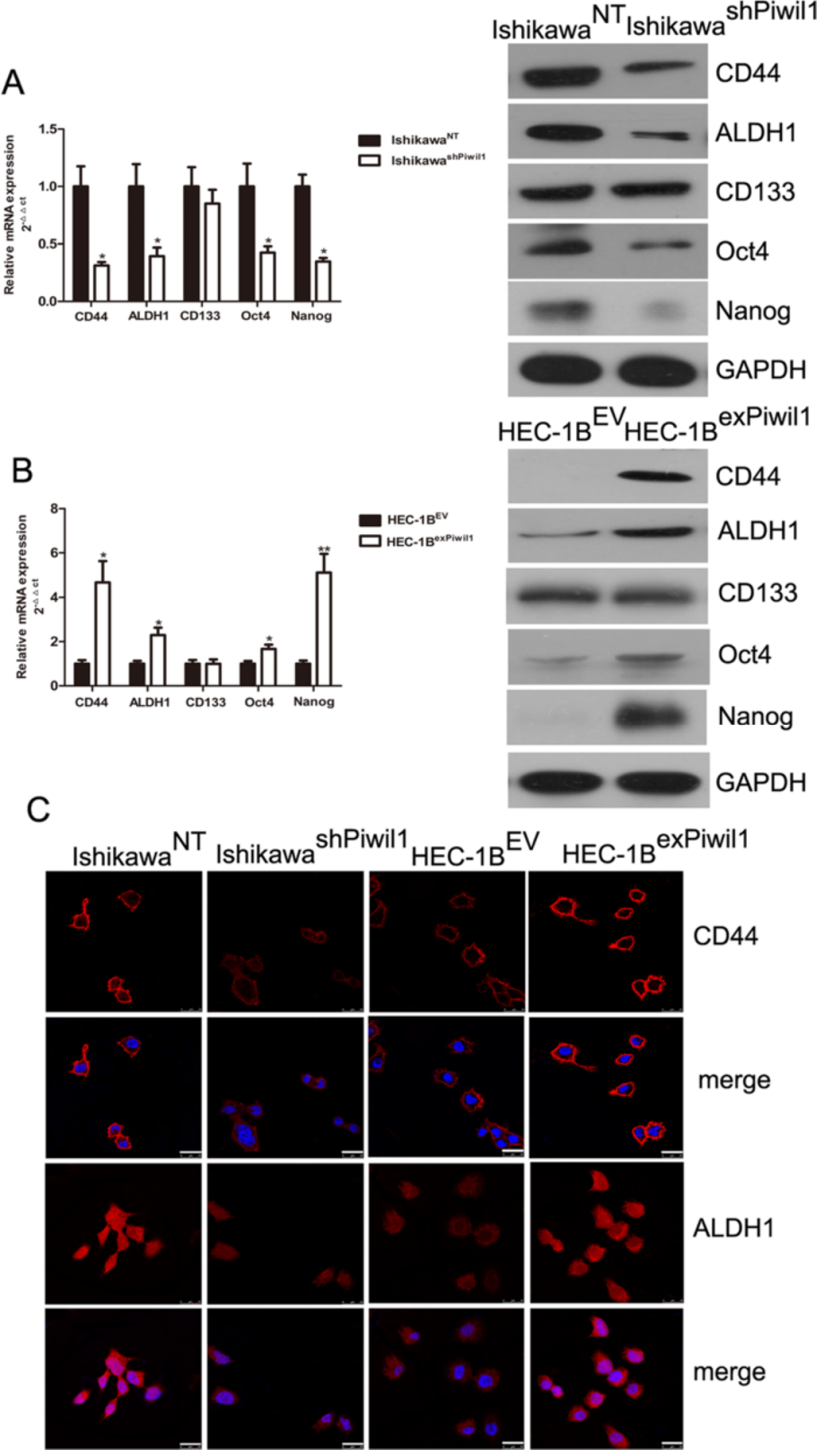
Piwil1 led to increased acquisition of endometrial cancer stem cell markers. **a** RT-qPCR and western blot demonstrated expression level of CD44, ALDH1, CD133, Oct4 and Nanog in Ishikawa^NT^ and Ishikawa^shPiwil1^ cells (*P < 0.05). **b** RT-qPCR and western blot demonstrated expression level of CD44, ALDH1, CD133, Oct4 and Nanog in HEC-1B^EV^ and HEC-1B^exPiwil1^ cells (*P < 0.05, **P < 0.01). **c** Representative immunofluorescence images showing CD44, ALDH1 and CD133 expression in Ishikawa^NT^ and Ishikawa^shPiwil1^ cells or in HEC-1B^EV^ and HEC-1B^exPiwil1^ cells. Nuclei were stained with DAPI. Scale bars, 25 μm

To evaluate the effect of Piwil1 in the acquisition of stem-like properties of endometrial cancer cells, we first studied whether the transfected cells created any shift in the patterns of expression of endometrial cancer stem cell markers, such as CD133, CD44 and ALDH1 [7, 22]. RT–qPCR, western blotting and immunofluorescence confirmed the downregulation of CD44 and ALDH1 in Ishikawa^shPiwil1^ cells relative to the control cells (*P < 0.05, Fig. 3a and c). For the HEC-1B^exPiwil1^ cells, we observed increased expression of CD44 and ALDH1 (*P < 0.05, Fig. 3b and c). There was no substantial difference in CD133 between transfected cells and control cells. Furthermore, we also observed decreased expression of standard stem cell markers, such as Oct4 and Nanog, in Ishikawa^shPiwil1^ cells and increased expression of Oct4 and Nanog in HEC-1B^exPiwil1^ cells and (*P < 0.05, ** *P* < 0.01, Fig. 3a and b).

### Piwil1 increased tumor growth potential

**Fig. 4:**
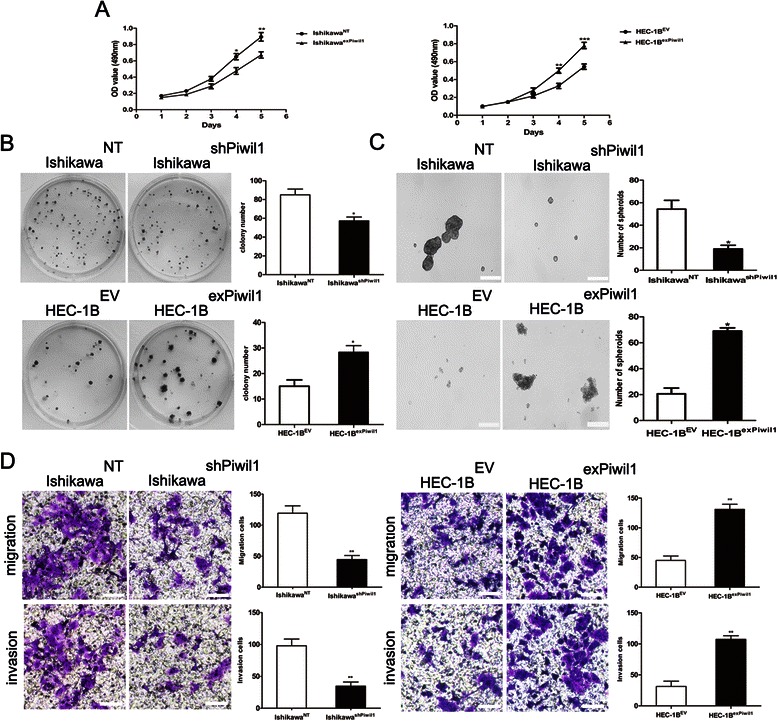
Piwil1 increased tumor growth potential and enhanced tumor metastatic potential. **a** The effects of Piwil1 on the cell growth of transfected cells were determined by MTT assay (*P < 0.05, **P < 0.01, ***P < 0.001). **b** Plated colony forming assays were performed to measure cell proliferation of transfected cells. Representative images are shown. The colonies were counted and graphed (*P < 0.05). **c** Representative images of spheroid colonies were shown. Scale bars, 100 μm. Quantification of the absolute number of spheroids formed by Ishikawa^shPiwil1^ cells or HEC-1B^exPiwil1^ cells compared to the control cells after 5 days (*P < 0.05). **d** Cell migration and invasion was measured in transwell chambers. Quantification of migration after 24 h and quantification of invasion after 48 h. Data are reported as fold induction vs control cells. Scale bar, 100 μm. **P < 0.01 Representative images are shown

Once we had demonstrated that Piwil1 was associated with the acquisition of stem-like features, we next considered whether this stem-like phenotype was associated with particular tumor biology properties. The most obvious approach was to assess its impact on tumor growth. We found that Ishikawa^shPiwil1^ cells showed a reduced viability as measured by MTT assay in comparison with Ishikawa^NT^ cells (*P < 0.05, ** *P* < 0.01, Fig. 4a) and HEC-1B^exPiwil1^ cells grew faster than control cells (** *P* < 0.01, *** *P* < 0.001, Fig. 4a). We also performed colony formation assays to further explore the role of Piwil1 in cell proliferation. We found more pronounced and frequent colony formation in Ishikawa^NT^ cells than was found in Ishikawa^shPiwil1^ cells (*P < 0.05, Fig. 4b). For HEC-1B^exPiwil1^ cells, the ability to form colonies was more pronounced than HEC-1B^EV^ cells (*P < 0.05, Fig. 4b). The tumorsphere assay can identify stem-like properties, as it highlights the cells’ capacity for self-renewal and ability to form a three-dimensional tumor-like sphere in vitro. The ability to form spheroid of Ishikawa^NT^ cells was more pronounced than Ishikawa^shPiwil1^ cells (*P < 0.05, Fig. 4c). We also found that HEC-1B^exPiwil1^ cells showed a significant increase in spheroid formation compared with control cells (*P < 0.05, Fig. 4c).

### Piwil1 enhanced tumor metastatic potential

**Fig. 5:**
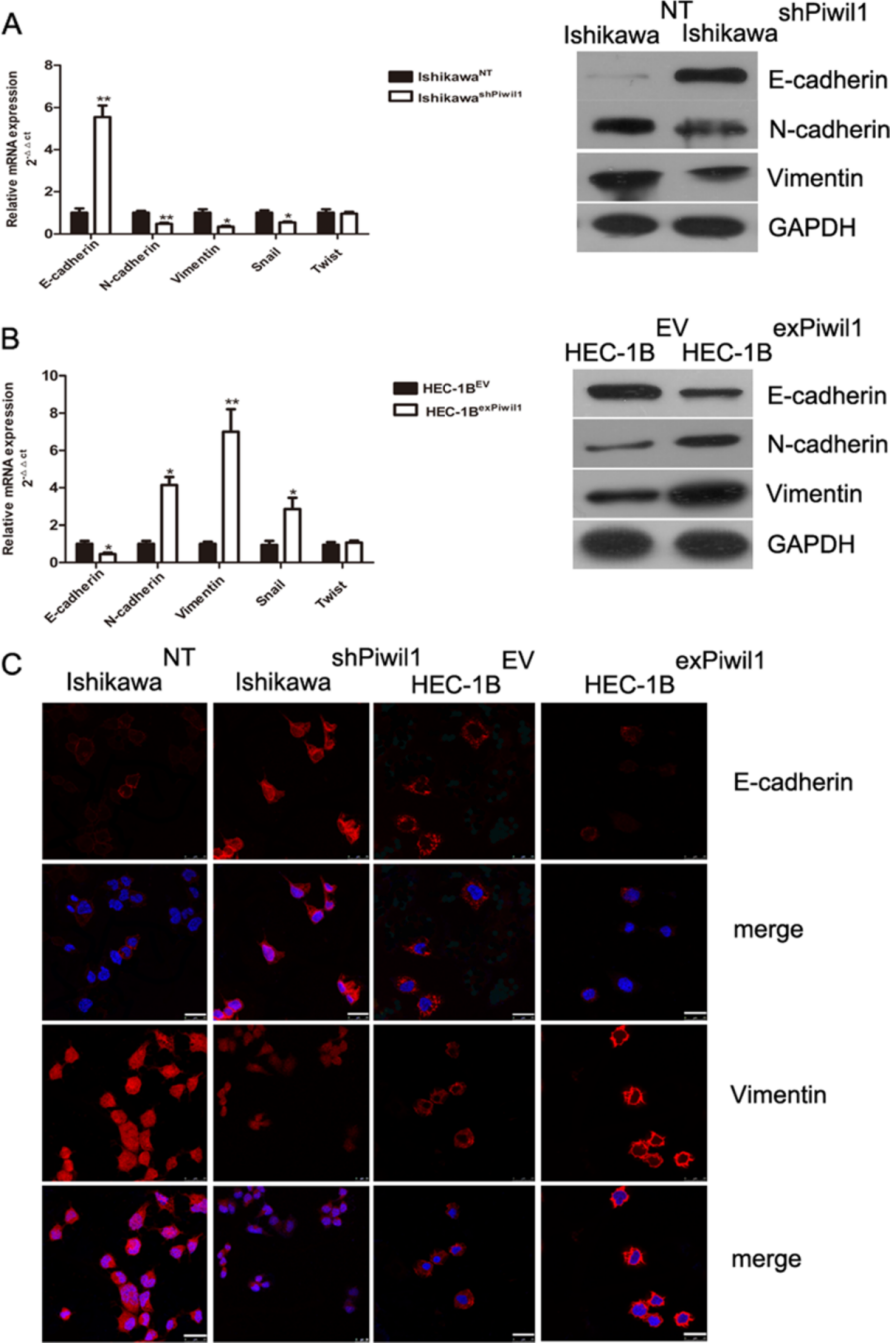
Piwil1 led to acquisition of mesenchymal markers and reduction of epithelial markers. **a** RT-qPCR and western blot demonstrated expression level of Vimentin, N-cadherin, E-cadherin, Snail and Twist in Ishikawa^NT^ and Ishikawa^shPiwil1^ cells (*P < 0.05, **P < 0.01). **b** RT-qPCR and western blot demonstrated expression level of Vimentin, N-cadherin, E-cadherin, Snail and Twist in HEC-1B^exPiwil1^ cells and HEC-1B^EV^ cells (*P < 0.05, **P < 0.01). **c** Representative images of immunofluorescence staining of EMT markers in Ishikawa^shPiwil1^ cells or HEC-1B^exPiwil1^ cells compared with control cells. Nuclei were stained with DAPI. Scale bars, 25 μm

Increased migration and invasion are another biology properties of CSCs. To assess whether Piwil1 affected migration and invasion of endometrial cancer cells, migration or invasion assays were performed. As indicated in Fig. 5a, migratory ability was significantly decreased in Ishikawa^shPiwil1^ cells (**P < 0.01), but enhanced in HEC-1B^exPiwil1^ cells (**P < 0.01, Fig. 4d). Consistent with the results of the migration assay, invasive ability was significantly decreased in Ishikawa^shPiwil1^ cells, but enhanced in HEC-1B^exPiwil1^ cells (**P < 0.01, Fig. 4d).

### Piwil1 led to acquisition of mesenchymal markers and reduction of epithelial markers

There is a link between EMT and the induction of the stem cell-like properties of cancer cells in solid tumors. We then examined the expression of EMT markers in transfected cells. RT–qPCR and western blotting were showed that epithelial marker E-cadherin were upregulated while the mesenchymal markers Vimentin and N-cadherin were downregulated in Ishikawa^shPiwil1^ cells (*P < 0.05, **P < 0.01, Fig. 5a). For HEC-1B^exPiwil1^ cells, E-cadherin were downregulated and Vimentin and N-cadherin were upregulated (*P < 0.05, **P < 0.01, Fig. 5b). Immunofluorescence staining further confirmed these results above (Fig. 5c). We also examined whether the expression of transcription factors which were closely associated with EMT were changed in transfected cells [23, 24]. Piwil1 could upregulate mRNA expression of Snail (*P < 0.01), while the mRNA level of Twist was not significantly changed (Fig. 5a and b).

### Endometrial cancer stem cell markers and EMT-related markers were detected in tumor-bearing mice

**Fig. 6:**
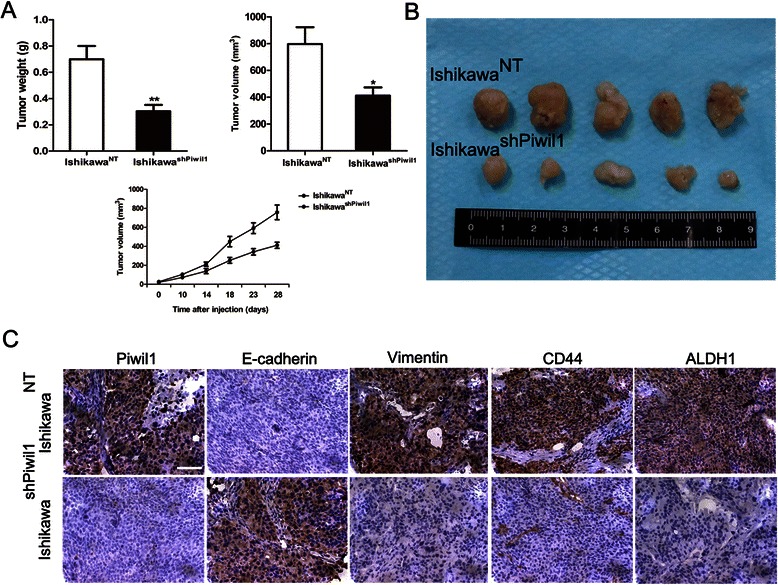
Endometrial cancer stem cell markers and EMT-related markers were detected in tumor-bearing mice. **a** and **b** Four weeks after injection of the indicated cells, tumors were removed and shown. The tumor weights and volumes were determined (*P < 0.05, **P < 0.01). Results represent tumor growth kinetics were also shown. **c** Representative Piwil1, E-cadherin, Vimentin, CD44 and ALDH1 immunohistochemical staining in tumor tissues generated from the Ishikawa^shPiwil1^ cells or Ishikawa^NT^ cells. Original magnification 400×, scale bar, 50 μm

Subcutaneous injection of Ishikawa^NT^ and Ishikawa^shPiwil1^ cells in immunodeficient mice demonstrated that the tumors generated from the Ishikawa^shPiwil1^ cells grew more slowly than those generated from the same amount of Ishikawa^NT^ cells (** *P* < 0.01, Fig. 6a). Then the expression of endometrial cancer cell markers and EMT-related markers was investigated by immunohistochemistry on tissues from tumor-bearing mice. Results revealed a significantly lower expression of Vimentin, CD44 and ALDH1 and a higher expression of E-cadherin in tumor tissues generated from the Ishikawa^shPiwil1^ cells compared with Ishikawa^NT^ cells (Fig. 6b).

## Discussion

One hypothesis related to carcinogenesis assumes that a specific population of tumor cells, CSCs, have the ability to initiate and maintain tumor growth [25]. A increasing body of evidence suggest that CSCs may originate not only from somatic stem cells (SSCs) where tumor suppressor genes could be silenced, but also from differentiated cells in which a self-renewal signaling pathway is activated [2].

Piwi, the other subclade of the Argonaute family, affected asymmetric division of stem cells in the Drosophila germline [26]. The early studies demonstrated that Piwi is essential for gametogenesis and is a key regulator of female germline stem cells [11, 12]. Four Piwi proteins are expressed in humans: Piwil1/Hiwi, Piwil2/Hili, Piwil3, and Piwil4/Hiwi2. Most studies showed that Piwi proteins are overexpressed in human cancers [26]. The expression of Piwil1, Piwil2, Piwil13, and Piwil4 were positively correlated with T stage, lymph node metastasis and clinical TNM and patients with higher expression had shorter survival time [27, 28]. In this study, we confirmed that the expression of Piwi proteins (Piwil1, Piwil2, Piwil13, and Piwil4) are different in endometrial cancer cell lines (Additional file 4) and we mainly focused on the role of Piwil1 in endometrial cancer.

It has been long recognized that the piwil1 is essential for stem cell self-renewal, division, spermatogenesis, RNA silencing, and translational regulation in *Drosophila*, mice and many other species [11, 29–31]. Piwil1 is also detected in human CD34^+^ cells but not in well-differentiated cells, suggesting it may determine or regulate human stem cell development [32]. However, reports focusing on effect of Piwil1 in tumor biology are limited, and the correlation between Piwil1 and endometrial cancer progression is not well documented. Here, we showed that Piwil1 was highly expressed in human endometrial cancer. Specific knockdown or overexpression of Piwil1 in endometrial cancer cell lines led to changes in tumor growth and metastatic potential in vitro and in vivo. Our results suggest that Piwil1 may be a part of the molecular pathway necessary for activating the cell’s capacity for self-renewal in endometrial cancer.

Although several studies have investigated the expression of Piwil1 in solid cancers, including endometrial cancer, this is the first study to address the function of Piwil1 in human endometrial cancer. We found that the normal endometrium produces weak levels of Piwil1, but that Piwil1 was extensively detected in endometrial cancer, which is in accordance with reports [14, 33]. Previous studies demonstrate that high levels of Piwil1 expression could increase the risk for tumor-related death [17, 18] and may be a poor prognostic factor for esophageal squamous cell carcinoma, gastric cancer and hepatocellular carcinoma [16, 27, 34]. Consistently, we found a significantly positive correlation between Piwil1 expression and lymphovascular space involvement, lymph node metastasis, varying depth of myometrial invasion and advanced disease stage, all of which are associated with high risk factors in endometrial cancer. Though these results are not sufficient to definitively designate Piwil1 as a prognostic factor for endometrial cancer, our expression data combined with our functional data suggest that Piwil1 might serve as a target for anticancer therapy.

To date, CSC markers of endometrial cancer have been identified, including CD133, CD44 and ALDH1 [7, 22, 35, 36]. Interestingly, we found that overexpression of Piwil1 could lead to increased acquisition of CD44 and ALDH1 and cells with knockdown of Piwil1 showed decreased expression levels of these markers. Besides expressing specific markers, CSCs are defined by their self-renewal capacity, their ability to generate a new tumor and an increased capacity for migration and invasion. In our study, we showed that the ability of proliferation, migration, invasion and sphere-forming in Ishikawa cells was decreased by Piwil1 knockdown. Conversely, HEC-1B cells overexpressing Piwil1 showed increased ability of proliferation, migration, invasion and sphere-forming. From our in vivo experiments, we discovered that the Piwil1 knockdown significantly inhibited the establishment of tumors. Therefore, we for the first time reported that Piwil1 may regulate stemness of endometrial cancer cells. Ishikawa cell line could be a useful model to study endometrial CSCs because it has a higher percentage of CD133^+^ cells and ability to undergo differentiation into other lineages [7]. However, CD133 showed no substantial difference between transfected cells and control cells. These findings raise the possibility that there are additional candidate genes that regulated CD133.

During the process of tumor metastasis, preceded by the EMT [37], metastasized cancer cells appear to require an ability to self-renew, similar to that seen in stem cells, to acquire a proliferative potential. In the present study, we presented several lines of evidence showing that Piwil1 was involved in EMT. We observed that Piwil1 could downregulate epithelial marker E-cadherin and upregulate mesenchymal markers Vimentin and N-cadherin in endometrial cancer cells. Snail plays a critical role in EMT [23]. Knock down of Snail could reverse EMT and significantly attenuated migration, invasion and cancer stem cell-like properties of cancer cells [38]. We further showed that Piwil1 could upregulate the transcription of Snail, indicating that Piwil1 may be required for Snail-induced EMT. However, further studies are required to identify the detailed mechanism involved in regulation of Piwil1 on Snail.

## Conclusions

In summary, to the best of our knowledge, we are the first to provide evidence that Piwil1 could induce EMT and endow endometrial cancer cells with stem-like properties. Thus, Piwil1 may represent a promising target for developing a novel treatment strategy for endometrial cancer.

## Additional files

Additional file 1: **List of primers used for RT-qPCR.** (DOC 40 kb)
Additional file 2: **The expression of Piwil1 in endometrial cancer lines.** (TIFF 3199 kb)
Additional file 3: **Human Cell Line Authentication.** (DOCX 300 kb)
Additional file 4: **The expression of PIWI proteins in endometrial cancer lines.** (TIFF 160 kb)

## Abbreviations

Piwil1: Piwi-like RNA-mediated gene silencing 1
shRNA: Small hairpin RNA
qPCR: Quantitative real-time reverse transcription polymerase chain reaction assays
FIGO: Federation International of Gynecology and Obstetrics
MTT: 3-(4,5-dimethyl-2-thiazolyl)-2,5-diphenyl-2-H-tetrazolium bromide
EMT: Epithelial-mesenchymal transition
IS: Immunoreactivity score
