# Supplementary material for: Stem cell protein Piwil1 endowed endometrial cancer cells with stem-like properties via inducing epithelial-mesenchymal transition
Source: BMC Cancer. 2015 Oct 27;15:811. doi: 10.1186/s12885-015-1794-8 (PMC4624602; doi:10.1186/s12885-015-1794-8)
Supplement: Additional file 1: — List of primers used for RT-qPCR. (DOC 40 kb) [file 12885_2015_1794_MOESM1_ESM.doc]

Additional file 1: Table S1. List of primers used for RT-qPCR

| Piwil1-Fw | TGTTGTCAAGTAATCGGA |
| --- | --- |
| Piwil1-Rev | GGCAATCTTTGTAGCAAT |
| CD44-Fw | CAACACAAATGGCTGGTACG |
| CD44-Rev | TCATCAATGCCTGATCCAGA |
| CD133- Fw | AATGACCCTCTGTGCTTGGT |
| CD133-Rev | GGATTGATAGCCCTGTTGGA |
| ALDH1-Fwd | GGAACAAATAAAGCCAAGTGC |
| ALDH1-Rev | CAAATCGGTGAGTAGGACAGG |
| Oct4-Fwd | GCGACTATGCACAACGAGAG |
| Oct4-Rev | GTGAAGTGAGGGCTCCCATA |
| Nanog-Fwd | GCAGTTCCAGCCAAATTCTC |
| Nanog-Rev | CACGTCTTCAGGTTGCATGT |
| E-cadherin-Fwd | TTGCTACTGGAACAGGGACAC |
| E-cadherin -Rev | CCCGTGTGTTAGTTCTGCTGT |
| N-cadherin -Fwd | GGAGACATTGGGGACTTCATT |
| N-cadherin -Rev | TCCTGCTCACCACCACTACTT |
| Vimentin-Fwd | TGCGTGAAATGGAAGAGAACT |
| Vimentin-Rev | TCAGGTTCAGGGAGGAAAAGT |
| GAPDH-Fwd | CAAATTTGGTCGTATTGGG |
| GAPDH-Rev | CTGGAAGATGGTGATGGGATT |
| Piwil2-Fw | TTGGCCTCAAGCTCCTAGAC |
| Piwil2-Rev | CATGCCACGGAACATGGAC |
| Piwil3-Fw | TGGGAATGTCATGTTTGCTGG |
| Piwil3-Rev | ACCACACACTGGCTTGGAAT |
| Piwil4-Fw | AGCAAAAGCATTCGACGGTG |
| Piwil4-Rev | ACGGGAGAACTTGATGGCAG |
