# Supplementary figures and images for: Stem cell protein Piwil1 endowed endometrial cancer cells with stem-like properties via inducing epithelial-mesenchymal transition
Source: BMC Cancer. 2015 Oct 27;15:811. doi: 10.1186/s12885-015-1794-8 (PMC4624602; doi:10.1186/s12885-015-1794-8)

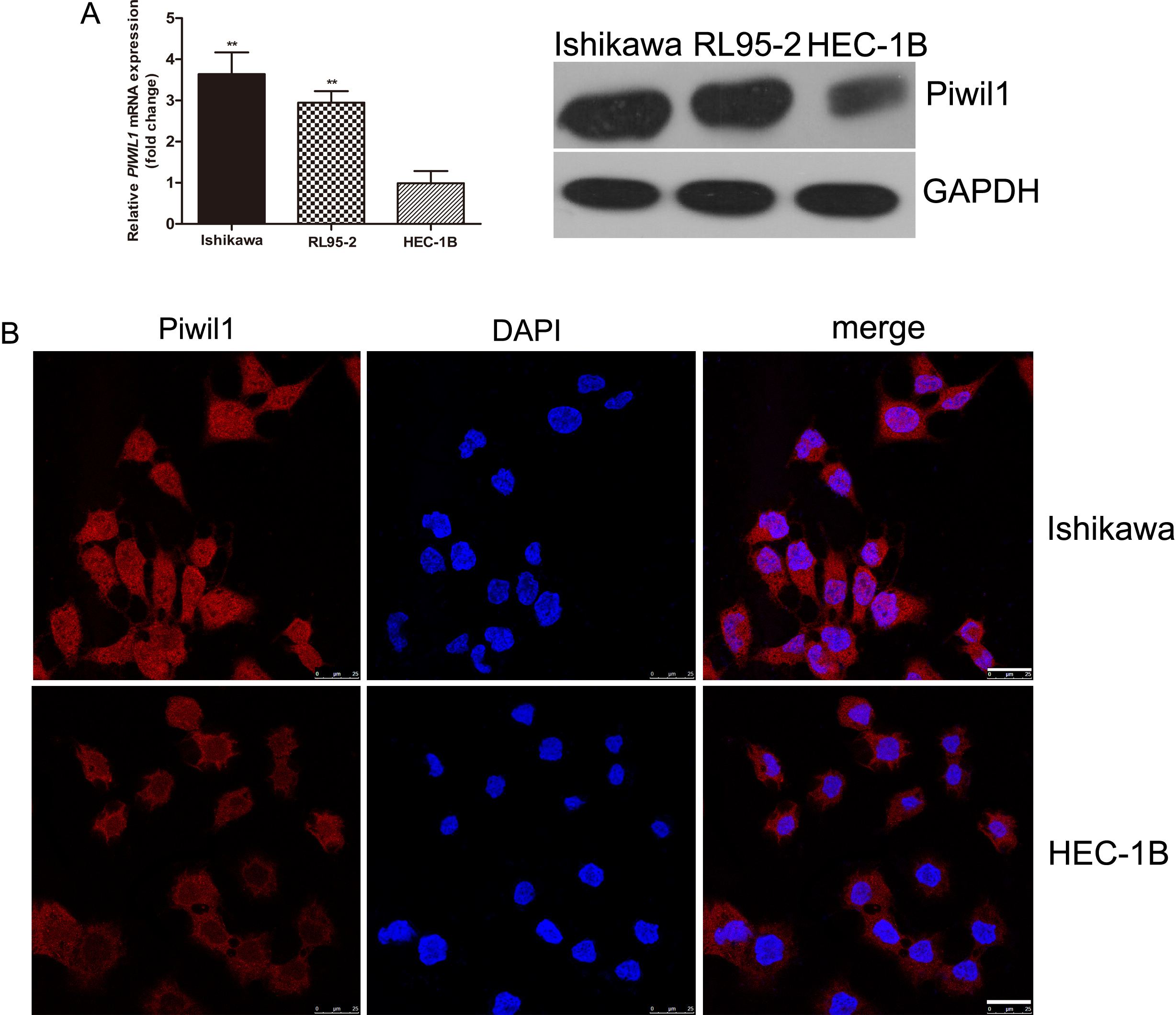

Supplement: Additional file 2: — The expression of Piwil1 in endometrial cancer lines. (TIFF 3199 kb) [file 12885_2015_1794_MOESM2_ESM.tiff]

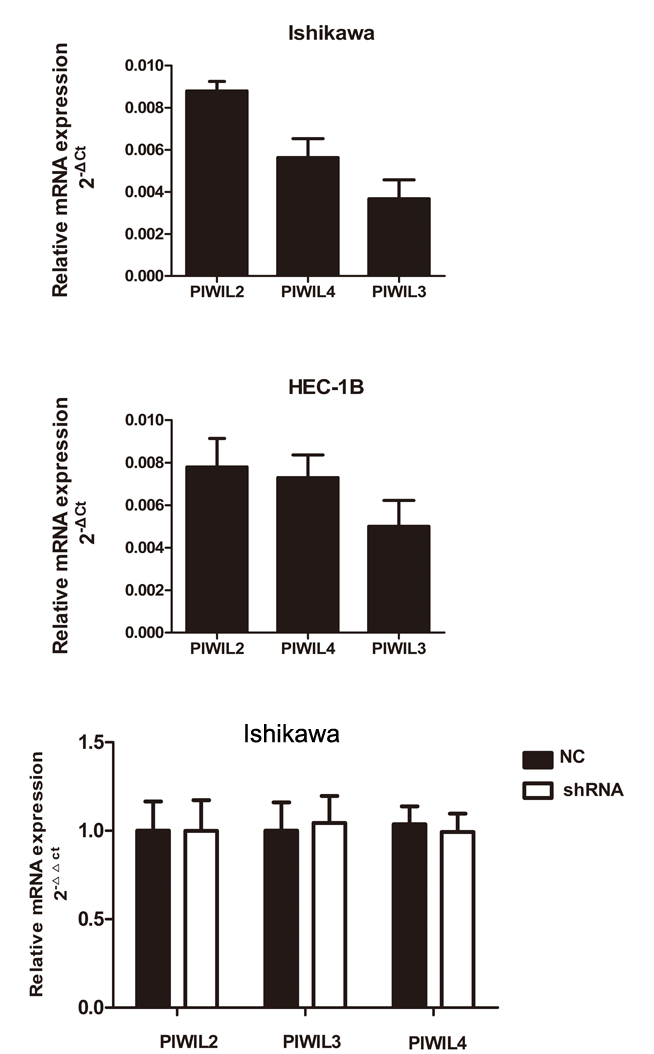

Supplement: Additional file 4: — The expression of PIWI proteins in endometrial cancer lines. (TIFF 160 kb) [file 12885_2015_1794_MOESM4_ESM.tiff]
