# Supplementary material for: Stem cell protein Piwil1 endowed endometrial cancer cells with stem-like properties via inducing epithelial-mesenchymal transition
Source: BMC Cancer. 2015 Oct 27;15:811. doi: 10.1186/s12885-015-1794-8 (PMC4624602; doi:10.1186/s12885-015-1794-8)

**Report of Human Cell Line Authentication**

Delivery Date: 2015-7-6

Analysis Date: 2015-7-10

Ⅰ. Samples

Three cell pellets samples received on 2015-7-6.

Sample Name: ‘Ishikawa’, [labeled](http://dict.cnki.net/dict_result.aspx?searchword=%e6%a0%87%e8%ae%b0&tjType=sentence&style=&t=labeled) as ‘001’;

Sample Name: ‘HEC-1B’, [labeled](http://dict.cnki.net/dict_result.aspx?searchword=%e6%a0%87%e8%ae%b0&tjType=sentence&style=&t=labeled) as ‘002’.

Ⅱ. Method and Procedure

1. PCR amplification with Goldeneye 20A Kit;

2. Electrophoresis of PCR products with the GeneticAnalyzer 3130XL.

Ⅲ. Results

1. The results of the negative and positive control match expectations in the test.

2. The STR profiles of the cell line samples are in the attached table and figures.

003: ① No cross-contamination of other human cells was found in this test. ②No 100% matched cell lines were found in the [ATCC, DSMZ or JCRB](http://cellbank.nibio.go.jp/cellbank_e.html) data banks.

Operator: Suhua Zhang Auditor: Shumin Zhao

Table 1: STR profiles of Ishikawa cell line

| Cell line 009（Figure. 001） | | |
| --- | --- | --- |
| Marker | Allele1 | Allele 2 |
| D19S433 | 12.2 | 14,15 |
| D5S818 | 10 | 11 |
| D21S11 | 27 | 28 |
| **D18S51** | **12** | **20,21,22** |
| D6S1043 | 12 | 18 |
| D3S1358 | 15 | 16 |
| D13S317 | 9 | 12 |
| D7S820 | 9 | 10 |
| D16S539 | 9 | 9 |
| **CSF1PO** | **11** | **12,13** |
| PentaD | 10 | 11 |
| Amelogenin | X | X |
| vWA | 14 | 17 |
| D8S1179 | 13 | 16 |
| TPOX | 8 | 8 |
| PentaE | 11 | 19 |
| TH01 | 9 | 10 |
| **D12S391** | **18** | **20,21** |
| D2S1338 | 19 | 21 |
| FGA | 21 | 22 |

Table 2: STR profiles of HEC-1B cell line

| Cell line 001（Figure. 003） | | |
| --- | --- | --- |
| Marker | Allele1 | Allele 2 |
| D19S433 | 13 | 13 |
| D5S818 | 11 | 13 |
| D21S11 | 30 | 31 |
| D18S51 | 16 | 20 |
| D6S1043 | 12 | 18 |
| D3S1358 | 15 | 15 |
| **D13S317** | **11(186.59)** | **16(207.13)** |
| D7S820 | 9 | 11 |
| D16S539 | 11 | 12 |
| CSF1PO | 10 | 12 |
| PentaD | 9 | 13 |
| Amelogenin | X | X |
| vWA | 18 | 18 |
| D8S1179 | 13 | 14 |
| TPOX | 8 | 11 |
| PentaE | 11 | 11 |
| TH01 | 6 | 7 |
| D12S391 | 18 | 19 |
| D2S1338 | 18 | 19 |
| FGA | 21 | 21 |

ECACC

Ishikawa


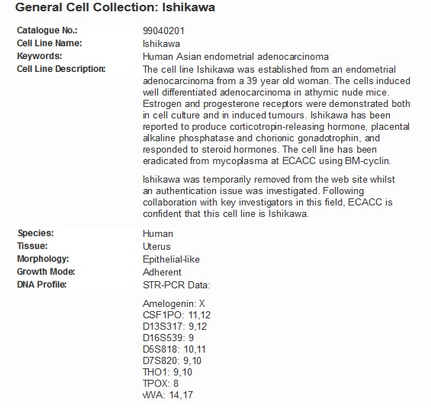


ATCC


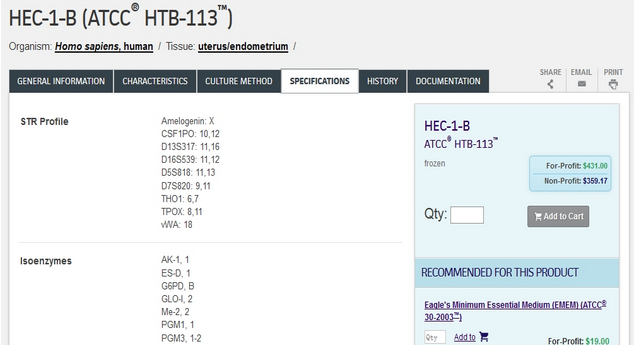

Supplement: Additional file 3: — Human Cell Line Authentication. (DOCX 300 kb) [file 12885_2015_1794_MOESM3_ESM.docx]
